# A novel Doppler echocardiographic score reflecting cardiac functional status can predict adverse outcome in acute myocardial infarction

**DOI:** 10.1007/s12574-012-0111-7

**Published:** 2012-02-11

**Authors:** Yamato Fukuda, Nobuo Fukuda, Satofumi Morishita, Hisanori Shinohara, Homare Yoshida, Osamu Yasuda, Yasushi Shimoe, Yoshiyuki Tamura

**Affiliations:** 1 Department of Cardiology and Clinical Research, National Hospital Organization Zentsuji Hospital, Senyu-cho 2-1-1, Zentsuji, Kagawa 765-0001 Japan; 2 Department of Cardiovascular Surgery, National Hospital Organization Zentsuji Hospital, Senyu-cho 2-1-1, Zentsuji, Kagawa 765-0001 Japan

**Keywords:** Tissue Doppler imaging, Acute myocardial infarction, Echocardiography, Cardiac function

## Abstract

**Background:**

*E*/*e*′ and *s*′ are thought to reflect left ventricular diastolic and systolic function, respectively. However, there are no reports on the combined use of *E*/*e*′ and *s*′ in predicting the outcome in acute myocardial infarction (AMI).

**Methods:**

For 20 months beginning in October 2006, we enrolled 65 AMI patients who had undergone Swan–Ganz (SG) catheterization and echocardiography just after reperfusion therapy. We measured the cardiac index (CI) and the pulmonary capillary wedge pressure (PCWP) via an SG catheter and determined routine echocardiographic indices, including transmitral flow velocity (*E*), mitral annulus velocities at systole (*s*′) and early diastole (*e*′), and *E*/*e*′. In addition, we rounded off the values of *s*′ (cm/s) and *E*/*e*′ (ratio of cm/s to cm/s) to the nearest integer, and designated them the *s*′-score and *E*/*e*′-score, respectively. We also defined the cardiac status score as the *s*′-score subtracted from the *E*/*e*′-score. In Study 1, we investigated the relationships between hemodynamic parameters (CI and PCWP) and echocardiographic indices, including the cardiac status score. In Study 2, we excluded patients with Killip class ≥II, yielding a final study population of 55 patients in whom we investigated whether the cardiac status score could predict adverse cardiac events.

**Results:**

Only the cardiac status score significantly correlated with both the PCWP and the CI. In the Cox proportional hazards model, significant predictors were the left ventricular ejection fraction (LVEF), estimated glomerular filtration rate (eGFR), and cardiac score ≥3.0.

**Conclusions:**

The novel score achieved in this study by subtracting the *s*′-score from the *E*/*e*′-score could be highly useful for predicting outcomes in AMI with Killip class I.

## Introduction

The values of *E*/*e*′ and *s*′, measured by pulse and tissue Doppler imaging, are thought to reflect left ventricular (LV) diastolic and systolic function, respectively [1–5]. Cardiac relaxation and contraction should be considered to be part of a continuous cycle [6]. Furthermore, both have recently been found to predict the prognosis of patients with cardiac disease [7–11]. However, there are no reports on the combined use of *E*/*e*′ and *s*′ in predicting outcomes in patients with acute myocardial infarction (AMI).

Therefore, we evaluated the practical implications of the combined use of *E*/*e*′ and *s*′ in patients with AMI. In addition, we investigated whether the combined use of *E*/*e*′ and *s*′ could predict prognosis after AMI.

## Materials and methods

### Patients (Study 1): study to investigate the relationship between hemodynamic parameters and echocardiographic indices

In 85 consecutive patients with AMI examined over 20 months beginning in October 2006, 65 patients (39 men; mean age 68 ± 15 years, range 26–90 years) who had undergone Swan–Ganz (SG) catheterization and echocardiography just after reperfusion therapy (emergency percutaneous coronary intervention, PCI) were enrolled to investigate the relationship between hemodynamic parameters and echocardiographic indices. AMI was defined by the following criteria: (1) chest pain ≥30 min in duration, (2) electrocardiographic ST junction elevation ≥0.1 mV in two or more leads in the same vascular territory, and (3) subsequent elevation of creatine phosphokinase (CK) to more than twice the normal range. All 65 patients underwent emergency PCI within 24 h after the onset of AMI. Patients with congenital heart disease, atrial fibrillation, history of coronary revascularization, pacemaker implantation, unsuccessful reperfusion, or unsatisfactory echocardiographic imaging were excluded.

### Patients (Study 2): study to investigate adverse outcomes of AMI

In addition, patients with heart failure of Killip class ≥II on admission were excluded to permit the investigation of adverse cardiac events, because these patients had already received aggressive treatment for heart failure on admission. Our final study population excluded ten of the aforementioned 65 patients, resulting in 55 patients without heart failure. All 55 patients were followed up after admission. Endpoints included all-cause death, heart failure, and revascularization. The 55 patients were divided into the following two groups based on the occurrence of events: E group (*n* = 13 patients), having events; and N group (*n* = 42 patients), having no events.

### Blood samples

Venous samples for measuring plasma brain natriuretic peptide (BNP) concentrations were obtained at the time of admission, before PCI. Plasma BNP concentrations were measured using a commercially available specific radioimmunoassay for human BNP (Shiono RIA BNP assay kit; Shionogi Co., Ltd., Tokyo, Japan). Venous samples for measuring the serum CK were obtained every 4 h until the CK levels peaked. The maximum CK was defined as the maximum CK concentration during hospitalization.

### Hemodynamic parameters

SG catheterization was performed just after PCI, and echocardiography was performed within 30 min after the completion of catheterization. Hemodynamic parameters, consisting of the cardiac index (CI) and the pulmonary capillary wedge pressure (PCWP), were measured by the SG catheter.

### Echocardiography

Echocardiography was performed using a Siemens Sequoia 512 ultrasound machine equipped with a sector transducer (carrier frequency of 2.5 MHz). The following routine echocardiographic parameters were measured just after PCI: left atrial diameter (LAD); LV end-diastolic volume index (LVEDVI); LV end-systolic volume index (LVESVI); LV ejection fraction (LVEF); peak early diastolic velocity (*E*) of LV inflow; peak systolic, early, and end-diastolic longitudinal velocity (*s*′, *e*′, and *a*′) of the mitral annulus; and the ratio of *E* to *e*′ (*E*/*e*′). In addition, we rounded off the values of *s*′ (cm/s) and *E*/*e*′ (ratio of cm/s to cm/s) to the nearest integer, and gave them unitless scores. A score of 3 was assigned to *s*′ when its value was between 0.1 and 2.4 cm/s, and a score of 20 when its value was above 20.5. Likewise, scores of 3 and 20 were assigned to *E*/*e*′ when its values were between 0.1 to 2.4, and above 20.5, respectively. We defined these scores as the *s*′-score and the *E*/*e*′-score, respectively. We also defined the cardiac status score as the value resulting from subtraction of the *s*′-score from the *E*/*e*′-score. The LAD was measured as the maximum dimension along the parasternal long-axis view from two-dimensionally guided M-mode tracings. LVEDVI and LVESVI were obtained using the modified biplane Simpson’s method from the apical four- and two-chamber views, normalized for body surface area, and LVEF was calculated by the following formula: (LVEDVI − LVESVI)/LVEDVI × 100 (%) [12]. The LV inflow velocity curve was obtained in the apical long-axis view with the pulsed Doppler sample volume positioned at the tips of the mitral leaflets during diastole [13]. The mitral annulus velocity was measured in the apical four-chamber view using pulsed Doppler tissue imaging by placing a sample volume at the lateral and septal portions of the mitral annulus. The average values of the lateral and septal annulus velocities were defined as the *s*′, *e*′, and *a*′ velocities.

### Study 1

The relationship between the hemodynamic parameters (CI and PCWP) measured just after PCI and echocardiographic indices performed just after PCI, including the status score, were investigated in the 65 patients in Study 1.

### Study 2

Patient characteristics, plasma BNP, and maximum CK levels (obtained during the hospital visit), hemodynamic parameters, and echocardiographic indices were compared between the two groups. We investigated whether the echocardiographic indices, including the cardiac status score measured just after PCI, could predict prognosis after AMI in the 55 patients in Study 2.

### Statistical analysis

Values were expressed as means ± standard deviations. A level of *p* < 0.05 was accepted as being statistically significant. Statistical analysis was performed with standard statistical packages (StatView 5.0 and SPSS 2.0).

Kolmogorov–Smirnov analysis was used to test for a normal distribution of the cardiac status score. Linear regression analysis was used to evaluate the relationship between echocardiac indices and hemodynamic parameters (PCWP and CI) in Study 1.

Significance between the two groups was evaluated with the unpaired *t*-test for continuous variables and the Chi-squared test for categorical variables. A log-rank test was used to analyze Kaplan–Meier survival curves. These curves determined the time-dependent, cumulative, cardiac event-free rates in patients who were stratified into two groups on the basis of the optimal cutoff value of the cardiac status score as determined by receiver operating characteristic (ROC) curve analysis. In addition, a Cox proportional hazards analysis was performed to evaluate the associations between cardiac events and various features. ROC curve analysis was conducted to illustrate various cutoff values of BNP, *E*/*e*′, and the cardiac status score for predicting cardiac events and to determine the optimal sensitivity and specificity. As ROC curve analysis could not be used to determine the cutoff values of LVEF, we used values that had previously been reported [14]. And we used the median values for the maximum CK and LAD with the Cox proportional hazard analysis.

## Results

### Study 1

#### Reproducibility of cardiac status score measurements

When the cardiac status scores of 40 subjects were recorded twice by the same observer, the measurements were well correlated (*r* = 0.98), with a mean percentage error of 2.0%. When two observers independently recorded the cardiac status scores in 20 subjects, the mean interobserver difference was 1 ± 1.

#### Relationship between hemodynamic parameters and echocardiographic variables (Table 1)

In this study, no patient had an *s*′ value ≤2.4 or ≥20.4 cm/s, and none had an *E*/*e*′ value ≤2.4 or ≥20.4 cm/s. Kolmogorov–Smirnov analysis showed a normal distribution of the cardiac status score (*p* < 0.05).

**Table 1:** Correlation of echocardiographic parameters with hemodynamic parameters in Study 1

	Simple linear regression analysis			
	PCWP		CI	
	Correlation coefficient (*r*)	*p*-value	Correlation coefficient (*r*)	*p*-value
*E*/*e*′	**0.33**	**<0.01**	−0.24	NS
*s*′	−0.15	NS	**0.40**	**<0.01**
Cardiac status score	**0.30**	**<0.05**	**−0.35**	**<0.01**

Bold values are statistically significant (*P* < 0.05)

*PCWP* pulmonary capillary wedge pressure, *CI* cardiac index, *E*/*e*′ the ratio of the peak early diastolic velocity of the left ventricular inflow to the peak velocity of the mitral annulus in early diastole, *s*′ peak velocity of the mitral annulus during systole, *Cardiac status score* scored *s*′ subtracted from scored *E*/*e*′

**Fig. 1:**
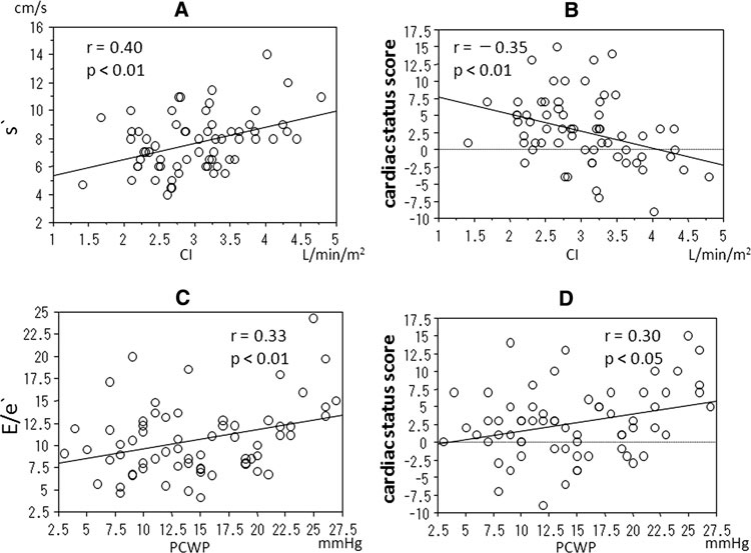
The cardiac index (CI) showed a significant positive correlation with *s*′ (**a**) and a significant negative correlation with the cardiac status score (**b**). The pulmonary capillary wedge pressure (PCWP) showed a significant positive correlation with *E*/*e*′ (**c**) and the cardiac status score (**d**)

The CI (3.02 ± 0.71 l/min/m^2^) showed a significant negative correlation with the PCWP (14.7 ± 6.1 mmHg) (*r* = 0.26, *p* < 0.05). The CI showed a significant positive correlation with *s*′ (*r* = 0.40, *p* < 0.01) (Fig. 1a) and *e*′ (*r* = 0.35, *p* < 0.01), and a significant negative correlation with the cardiac status score (*r* = −0.35, *p* < 0.01) (Fig. 1b). The CI showed a non-significant positive correlation with LVEDVI (*r* = 0.25, *p* = 0.06), LVESVI (*r* = 0.23, *p* = 0.09), and a non-significant negative correlation with *E*/*e*′ (*r* = −0.24, *p* = 0.06). However, there were no significant correlations between the CI and other echocardiographic variables, including LVEF.

The PCWP showed a significant positive correlation with *E*/*e*′ (*r* = 0.33, *p* < 0.01) (Fig. 1c) and the cardiac status score (*r* = 0.30, *p* < 0.05) (Fig. 1d). There were no significant correlations between the PCWP and other echocardiographic variables.

### Study 2

#### Baseline clinical characteristics and hemodynamic parameters (Table 2)

During the follow-up period (mean 201 ± 100 days), three patients died due to sudden cardiac death and ten patients had congestive heart failure. There were no revascularization events in this study. Two sudden deaths occurred within 30 days and one other occurred 105 days after follow-up began. Age, BNP level, creatinine level, and estimated glomerular filtration rate (eGFR) [15] were significantly higher in the E group compared to the N group. The PCWP was non-significantly higher in the E group compared to the N group. There were no significant differences between the groups in terms of blood pressure, hemoglobin A1c (HbA1c), and maximum CK.

**Table 2:** Comparison of patients’ baseline clinical characteristics and hemodynamic parameters in Study 2

	E group (*n* = 13)	N group (*n* = 42)	*p*-value
Age (years)	**75** **±** **11**	**66** **±** **15**	**<0.05**
Gender (male/female)	18/23	11/18	NS
Systolic BP (mmHg)	129 ± 23	130 ± 24	NS
Diastolic BP (mmHg)	68 ± 14	72 ± 15	NS
HR (bpm)	**78** **±** **16**	**73** **±** **15**	**<0.05**
Max. CK (mg/dl)	3234 ± 2486	2638 ± 2122	NS
BNP (pg/ml)	**282** **±** **237**	**134** **±** **164**	**<0.05**
Cre (mg/dl)	**1.1** **±** **0.4**	**0.8** **±** **0.3**	**<0.05**
eGFR	**53** **±** **20**	**74** **±** **24**	**<0.01**
HbA1c (%)	6.1 ± 1.1	6.0 ± 1.5	NS
LDL-cho (mg/dl)	119 ± 25	122 ± 32	NS
CI (L/min/m^2^)	3.2 ± 0.7	3.0 ± 0.7	NS
PCWP (mmHg)	18 ± 11	14 ± 5	0.06

Bold values are statistically significant (*P* < 0.05)

*BP* blood pressure, *HR* heart rate, *CK* creatine phosphokinase, *BNP* brain natriuretic peptide, *Cre* creatinine, *eGFR* estimated glomerular filtration rate, *HbA1c* hemoglobin A1c, *LDL-cho* low-density lipoprotein cholesterol, *CI* cardiac index, *PCWP* pulmonary capillary wedge pressure

#### Echocardiographic data (Table 3)

LVEF and *e*′ were significantly lower in the E group than in the N group. LVEDVI, LVESVI, *E*/*e*′, the *E*/*e*′-score, and the cardiac status score were significantly higher in the E group than in the N group. No other parameters showed significant differences between the groups.

**Table 3:** Comparison of echocardiographic parameters in Study 2

	E group (*n* = 13)	N group (*n* = 42)	*p*-value
LVEDVI (ml/m^2^)	**57** **±** **14**	**47** **±** **9**	**<0.01**
LVESVI (ml/m^2^)	**30** **±** **13**	**21** **±** **5**	**<0.01**
LVEF (%)	**49** **±** **11**	**57** **±** **7**	**<0.01**
LAD (mm)	**39** **±** **8**	**34** **±** **6**	**<0.05**
*E* (cm/s)	66 ± 18	59 ± 16	NS
*A* (cm/s)	79 ± 28	70 ± 22	NS
*E*/*A*	0.9 ± 0.3	0.9 ± 0.4	NS
*s*′ (cm/s)	7.1 ± 1.8	7.9 ± 2.0	NS
*e*′ (cm/s)	**5.3** **±** **1.2**	**6.9** **±** **2.2**	**<0.05**
*a*′ (cm/s)	9.0 ± 2.8	9.7 ± 2.6	NS
*E*/*e*′	**12.8** **±** **3.9**	**9.2** **±** **2.9**	**<0.01**
*s*′-score	7 ± 2	8 ± 2	NS
*E*/*e*′-score	**13** **±** **4**	**9** **±** **3**	**<0.01**
Cardiac status score	**6** **±** **5**	**1** **±** **5**	**<0.01**

Bold values are statistically significant (*P* < 0.05)

*LVEDVI* left ventricular end-diastolic volume index, *LVESVI* LV end-systolic volume index, *LVEF* left ventricular ejection fraction, *LAD* left atrial dimension, *LAVI* left atrial volume index, *E* peak early diastolic velocity of the mitral inflow, *A* peak late diastolic velocity of the mitral inflow, *s*′ peak systolic longitudinal velocity of the mitral annulus, *e*′ peak early diastolic longitudinal velocity of the mitral annulus, *a*′ peak late diastolic longitudinal velocity of the mitral annulus, *E*/*e*′ the ratio of *E* to *e*′, *s*′-score scored *s*′, *E*/*e*′-score scored *E*/*e*′, *Cardiac status score* *s*′-score subtracted from *E*/*e*′-score

#### Prognosis of subjects determined by univariate and multivariate Cox proportional hazards analyses (Tables 4 and 5)

We used univariate Cox proportional hazards analysis to determine the relationships between cardiac events and basal characteristics, blood-related parameters (BNP and maximum CK levels), and echocardiographic variables (Table 4). A cardiac status score greater than or equal to 3 proved to be a significant variable (hazard ratio 5.41, 95% confidence interval [CI] 1.49–19.70; *p* < 0.05). Furthermore, age, LVEF, and eGFR were significantly related to adverse outcomes, but *E*/*e*′ was not.

**Table 4:** Univariate Cox proportional hazards analysis for adverse events

Variables	Univariate Cox proportional hazards model		*p*-value
	Hazard ratio	95% confidence interval	
Age, per-year increase	**1.055**	**1.001–1.112**	**<0.05**
LVEF ≤45%	**4.395**	**1.425–13.549**	**<0.05**
LAD ≥35 mm	1.376	0.462–4.094	NS
*E*/*e*′ ≥11	2.951	0.990–8.792	0.05
Cardiac status score ≥3.0	**5.412**	**1.487–19.700**	**<0.05**
Max. CK ≥2400 mg/dl	1.121	0.627–2.001	NS
BNP ≥140 pg/ml	2.812	0.864–9.149	NS
eGFR ≥60 ml/min	**5.387**	**1.478–19.630**	**<0.05**

Bold values are statistically significant (*P* < 0.05)

*LVEF* left ventricular ejection fraction, *LAD* left atrial dimension, *E*/*e*′ the ratio of *E* to *e*′, *Cardiac status score* scored *s*′ subtracted from scored *E*/*e*′, *CK* creatine phosphokinase, *BNP* brain natriuretic peptide, *eGFR* estimated glomerular filtration rate

**Table 5:** Multivariate Cox proportional hazards analysis for adverse events

Variables	Multivariate Cox proportional hazards model		*p*-value
	Hazard ratio	95% confidence interval	
Age, per-year increase	1.052	0.973–1.139	NS
LVEF ≤45%	**4.583**	**1.075–19.550**	**<0.05**
LAD ≥35 mm	3.894	0.974–15.571	NS
Cardiac status score ≥3.0	**5.128**	**1.060–24.810**	**<0.05**
Max. CK ≥2400 mg/dl	1.681	0.400–7.068	NS
BNP ≥140 pg/ml	0.748	0.115–4.849	NS
eGFR ≤60 ml/min	**7.460**	**1.123–49.541**	**<0.05**

Bold values are statistically significant (*P* < 0.05)

*LVEF* left ventricular ejection fraction, *LAD* left atrial dimension, *E*/*e*′ the ratio of *E* to *e*′, *Cardiac status score* scored *s*′ subtracted from scored *E*/*e*′, *CK* creatine phosphokinase, *BNP* brain natriuretic peptide, *eGFR* estimated glomerular filtration rate

With the multivariate Cox proportional hazards analysis, LVEF, eGFR, and the cardiac status score were independent predictors of adverse outcomes, as shown in Table 5.

#### ROC curve analysis of cardiac status scores and Kaplan–Meier survival curves (Fig. 2)

**Fig. 2:**
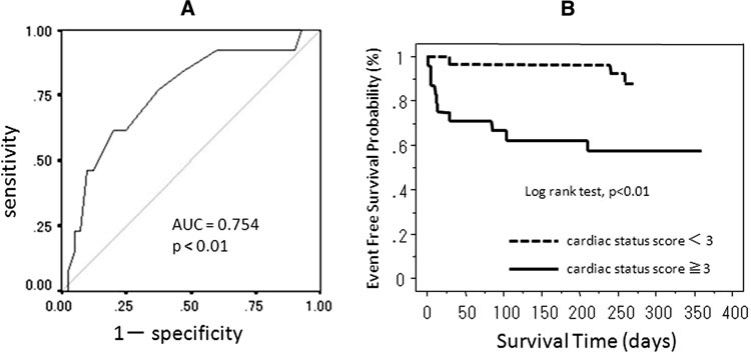
Receiver operating characteristic (ROC) curves for the cardiac status score. The area under the ROC curve (AUC) of the status index was 0.754 (**a**). Kaplan–Meier analysis for all adverse outcomes in patients with high or low cardiac status scores (**b**)

ROC curve analysis (Fig. 2a) indicated that the optimal cutoff value (3) for the cardiac status score had 77% sensitivity and 68% specificity for predicting cardiac events (area under the ROC curve [AUC] = 0.754, *p* < 0.01). The AUC for *E*/*e*′ was smaller (0.650) than that of the cardiac status score (not shown).

The Kaplan–Meier analysis showed that the group with cardiac status scores equal to or greater than 3 showed a significantly higher event rate than the group with cardiac status scores less than 3 (*p* < 0.01) (Fig. 2b).

## Discussion

The three basic events in the cardiac cycle are: (1) LV contraction, (2) LV relaxation, and (3) LV filling. The energy generated during systole is stored within the myocardium, and following myocyte relaxation, the ventricle uncoils, creating LV suction. Therefore, LV contractility contributes to LV relaxation. Oki et al. [5] showed that the systolic longitudinal velocity of the mitral annulus by tissue Doppler imaging correlated significantly with the peak d*P*/d*t* of the LV. Therefore, *s*′ may reflect the contractility and viability of the LV muscle after AMI. Moreover, Oki et al. [16] reported that the early diastolic longitudinal velocity of the mitral annulus showed a significant negative correlation with the isovolumic relaxation rate constant (tau) of the LV, and it did not depend on preload. *E*/*e*′ is an established index that indicates LV filling pressure and is a predictor of the prognosis of patients with diverse heart diseases [1–3, 7, 9, 11]. Because the cardiac status score reflects LV contractility, LV filling, and a portion of LV relaxation, it was hypothesized that this score might provide a measure of general cardiac function.

In the calculation of the cardiac status score, the *s*′-score and the *E*/*e*′-score were assumed to be simple, unitless scores. The cardiac status score could suggest the presence of a functional cardiac disorder, because a high cardiac status score indicates high PCWP and/or low cardiac output. It is unsurprising, therefore, that the majority of the events in patients with cardiac status score ≥3 occurred within 30 days in our study.

It was recently reported that an index combining diastolic and systolic tissue Doppler parameters (*E*/*e*′ divided by *s*′) could better predict LV end-diastolic pressure than other parameters, for example, *E*/*e*′ [17]. A high LV end-diastolic pressure indicates LV dysfunction and LV disorder. Therefore, the current study does not contradict the above-mentioned results. However, *E*/*e*′ divided by *s*′ was not a significant predictor of cardiac adverse outcomes in this study. This may be because, in this study, the cardiac status score was a significant predictor of the CI and the PCWP, whereas *E*/*e*′ divided by *s*′ was not.

Other recent studies found that renal function was an important factor in predicting adverse outcomes in various cardiac diseases [18]. Our present research on predicting adverse outcomes in AMI patients agrees with these results.

It has been reported that the BNP level is an important factor in predicting adverse outcomes in AMI; however, we did not find this to be the case [19, 20]. This may be because the mechanism underlying the BNP rise following AMI is complicated, and BNP values vary depending on the time after AMI onset [21]. One possible explanation for our findings is that, in this study, we determined BNP levels at the time of admission, before PCI. These levels might, therefore, be lower than in previous studies. Our present study suggests that the cardiac status score could be a better predictor of adverse outcomes than the BNP level, not only for the long term, but also during the period just after PCI.

Hillis et al. [9] and other groups [22–24] have reported that *E*/*e*′ is a significant predictor in AMI patients, whereas this was not the case in this study. We found that the cardiac status score was superior compared to *E*/*e*′. This may be because the cardiac status score reflected not only the CI but also the PCWP, whereas *E*/*e*′ reflected only the PCWP. In addition, we excluded patients with a Killip class equal to or greater than II, and performed echocardiography during the acute phase, just after PCI, and evaluated the adverse outcomes from admission onwards. Compared to *E*/*e*′, the cardiac status score could be a more useful index for predicting adverse events in AMI patients with Killip class I, both during the acute phase and in the long term.

In clinical settings, especially in cases of AMI, a simpler and easier score is needed. The cardiac status score that we newly defined in this study can be measured more easily, even if the patient is in an intensive care unit just after PCI for AMI.

Our present study suggests that, if the cardiac status score just after AMI is ≥3, we should closely observe the state of the patient and perform more active preventive therapies, such as the administration of human atrial natriuretic peptide (hANP) or a β-blocker.

### Limitations

This study has a few limitations. First, our study used a small population compared to previous studies [9, 22–24]. In the future, a larger study comparing the cardiac status score with other echocardiographic features is needed. The second limitation is the influence of the culprit lesion on the velocity of the mitral annulus. We adopted the mean value of the lateral and septal mitral annulus velocities to avoid that influence. However, in the future, studies using the two-dimensional speckle tracking method or three-dimensional echocardiography are needed. Finally, our study did not investigate the influence of the administration of β-blockers, hANP [25], or statins [26]. We administered nicorandil, renin–angiotensin system inhibitors, and statins to all patients enrolled. We also administered a β-blocker to patients who required it based on existing guidelines and hANP to patients with heart failure. However, in the future, further investigation of the influence of these drugs is needed.

## Conclusions

The left ventricular ejection fraction (LVEF), estimated glomerular filtration rate (eGFR), and the novel score obtained by subtracting scored *s*′ from scored *E*/*e*′ might be very useful in predicting adverse outcomes following acute myocardial infarction (AMI) in patients with Killip class I.
